# *De novo* synthesis of *trans*-10, *cis*-12 conjugated linoleic acid in oleaginous yeast *Yarrowia Lipolytica*

**DOI:** 10.1186/1475-2859-11-51

**Published:** 2012-07-04

**Authors:** Baixi Zhang, Chunchi Rong, Haiqin Chen, Yuanda Song, Hao Zhang, Wei Chen

**Affiliations:** 1 State Key Laboratory of Food Science and Technology, Jiangnan University, Wuxi, 214122, People's Republic of China; 2 School of Food Science and Technology, Jiangnan University, Wuxi, 214122, People's Republic of China

**Keywords:** Conjugated linoleic acid, Linoleate isomerase, Codon optimization, Multi-copy integration, *Propionibacterium acnes*, *Yarrowia lipolytica*

## Abstract

**Background:**

Conjugated linoleic acid (CLA) has many well-documented beneficial physiological effects. Due to the insufficient natural supply of CLA and low specificity of chemically produced CLA, an effective and isomer-specific production process is required for medicinal and nutritional purposes.

**Results:**

The linoleic acid isomerase gene from *Propionibacterium acnes* was expressed in *Yarrowia lipolytica* Polh. Codon usage optimization of the PAI and multi-copy integration significantly improved the expression level of PAI in *Y. lipolytica.* The percentage of *trans*-10, *cis*-12 CLA was six times higher in yeast carrying the codon-optimized gene than in yeast carrying the native gene. In combination with multi-copy integration, the production yield was raised to approximately 30-fold. The amount of *trans*-10, *cis*-12 CLA reached 5.9% of total fatty acid yield in transformed *Y. lipolytica*.

**Conclusions:**

This is the first report of production of *trans*-10, *cis*-12 CLA by the oleaginous yeast *Y. lipolytica*, using glucose as the sole carbon source through expression of linoleic acid isomerase from *Propionibacterium acnes*.

## Background

Conjugated linoleic acid (CLA) is a generic term used to describe a mixture of positional and geometric isomers of linoleic acid (LA) with conjugated double bonds. In the past three decades, CLA has attracted much attention because of its biologically beneficial functions. These include anti-carcinogenic, anti-atherogenic, anti-diabetic, anti-inflammatory and anti-obesity properties in animal models and humans. To date, three CLA isomers have been demonstrated to possess beneficial effects individually: *cis*-9, *trans*-11 CLA, *trans*-10, *cis*-12 CLA and *trans*-9, *trans*-11 CLA. Different isomers have different effects on metabolism and act through different cell signaling pathways [1-3].

In nature, CLA isomers occur in meat and dairy products derived from ruminants as a minor component of the lipid fraction [4]. Today, CLA, as a dietary supplement,is generally made from the LA of safflower and sunflower oils through alkaline isomerization in which additional functionally undetermined isomers are formed simultaneously [5,6]. Moreover, in the vast majority of the studies about CLA effectiveness on human volunteers, CLA mixtures rather than pure isomers have been used for supplementation and the results were often controversial. Therefore, to meet the requirements for medicinal and nutritional purposes, CLA isomers need to be biologically safe and highly specific. Biological production of CLA may be crucial to address these issues. It is well known that LA isomerase catalyzes the conversion of LA into CLA isomers. As early as 1965, Tove and colleagues demonstrated the existence of LA isomerase activity of producing *cis*-9, *trans*-11 CLA from LA in anaerobic rumen bacterium *Butyrivibrio fibrisolvens*[7]. So far, only three LA isomerases derived from *Lactobacillus reuteriClostridium sporogenes* and *Propionibacterium acnes* have been fully characterized*.* The first two isomerases are *cis*-9, *trans*-11-CLA-producing isomerases and the other is a *trans*-10, *cis*-12-CLA-producing isomerase [8,9]. The LA isomerase derived from *P. acnes* (PAI) is the only LA isomerase with its crystal structure being characterized [10], and had been expressed in *Escherichia. coli*[11], *Saccharomyces. cerevisiae*, tobacco seed [12], rice [13] and *Lactococcus lactis* successively [14].

Unlike other enzymes such as fatty acyl desaturase and fatty acyl elongase, PAI use free fatty acids as the only substrate. Therefore, neither phosphatidylcholine, nor methyl-, CoA-, or triacylglycerol- esters of LA can be accepted as substrates [12]. Unfortunately, the amount of free fatty acid is low in most eukaryotic organisms, which thus becomes the primary limiting factor for CLA production by biological synthesis [12,13]. However, as an exception, *Y. lipolytica*, an oleaginous yeast, is able to accumulate significant quantities of free fatty acid within the cell [15]. Moreover, it can accumulate large amount of lipids (> 25% of cell dry weight) with a high proportion of LA in the fatty acid composition [16]. Therefore, the non-conventional oleaginous yeast *Y. lipolytica* was used as a model in this study, to investigate the production of *trans*-10, *cis*-12-CLA from endogenous LA by over-expressing the functional LA isomerase from *P. acnes*.

## Results

### PAI gene codon optimization

**Figure 1:**
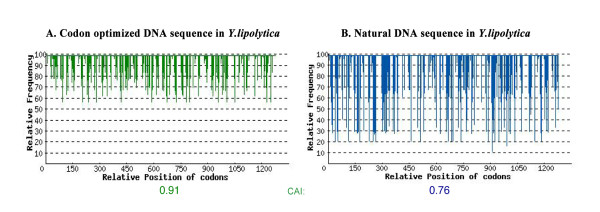
**Codon usage optimization of bacterial PAI gene for expression in*Y. lipolytica*.** Shown is the distribution of codon usage frequency along the coding sequence and the calculated codon adaptation index (CAI).

As the codon usage of the native *PAI* sequence differs from that preferred by *Y. lipolytica*, a *de novo* codon-optimized version of the gene was designed and synthesized. In this optimized version of the gene, 48% of all codons were replaced by *Y. lipolytica*-favored codons, and the AT/GC ratio was adjusted to that of the host. The codon adaption index (CAI) was improved from 0.76 (natural sequence) to 0.91 (synthetic gene) (Figure 1). Additionally, the Kozak element was added before the first AUG codon to prevent the leaky scanning of the ribosome. The translation product of the synthetic gene had the same amino acid sequence as native *PAI*. To test the effectiveness of codon optimization in the expression of the PAI, both the native sequence (*PAI*) and the codon-optimized sequence (*oPAI*) were employed for expression in *Y. lipolytica*.

### Construction of recombinant yeast strains

The mono-copy vector pINA1312 and the multi-copy vector pINA1292 were used for PAI expression in *Y. lipolytica*. pINA1312 contains the non-defective *ura3d1* gene for mono-copy expression, while pINA1292 contains the defective *ura3d4* gene, which is required in multiple copies to alleviate the uracil auxotrophy of the host [17]. Transformants with the non-defective vectors appeared on YNBD after 2-3 days of incubation, while transformants with defective vectors were observed after 4-6 days of incubation. Single-colony isolates were named according to strain, plasmid, gene and clone number as follows: Polh-1312-*PAI*-1 represents strain Polh, plasmid pINA1312, gene *PAI* and clone number 1. Seven Polh-1312-*PAI* transformants, twelve Polh-1312-*oPAI* transformants and thirteen Polh-1292-*oPAI* transformants were picked randomly and confirmed to be all positive by PCR analysis.

### *PAI* copy numbers in *Y. lipolytica* transformants

**Figure 2:**
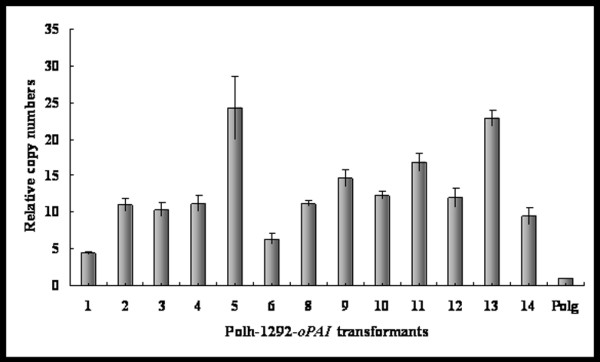
**Relative copy numbers of integrated expression cassettes in*Yarrowia lipolytica*multi-copy transformants.** Real-time PCR was used to estimate the copy number of the integrated expression cassettes among 13 Polh-1292-*oPAI* transformants. *Y. lipolytica* Polg was used as a control organism with a single copy of both the *URA3* and *SUC2* target sequences. As *URA3* and *oPAI* coexisted in expression cassette, the copy numbers of both genes were considered as equal.

The copy numbers of the integrated expression cassette among multi-copy transformants were estimated using the data obtained after RT-PCR analysis. *Y. lipolytica* Polg was used as a control organism with a single copy of both the *URA3* and *SUC2* target sequences. As *URA3* and *oPAI* coexisted in expression cassette, the copy numbers of both genes were considered as equal. Distribution of *PAI* copy numbers analyzed in 13 isolated Polh-1292-*oPAI* transformants is shown in Figure 2. For all the clones tested, copy numbers were in the range of 6-24 copies, having average12-13 copies/cell. The majority of the transformants (9 clones) contained 10-16 copies/cell. The highest copy number was 24 in strain Polh-1292-*oPAI*-5.

### Heterologous expression of PAI in *Y. lipolytica*

**Figure 3:**
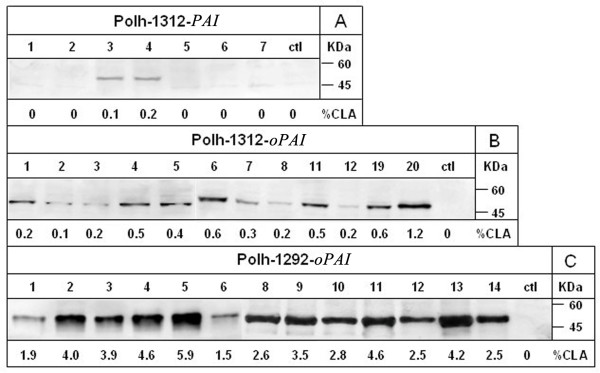
**Western blot analysis of recombinant PAI and percentage of CLA of total fatty acids (w/w).(a)** Western blot analysis of Polh-1312-*PAI* transformants, **(b)** Western blot analysis of Polh-1312-*oPAI* transformants and **(c)** Western blot analysis of Polh-1292-*oPAI* transformants. Numbers above the lines represents the individual transformants and control is the strain transformed with the empty vector pINA1312 or pINA1292. Molecular size markers are indicated on the right. The standard deviations were <1% of the values shown.

The expression level of the recombinant PAI in these yeast transformants was analyzed by Western blot using specific polyclonal antibodies raised against the recombinant PAI from *E. coli*. The recombinant PAI was present in all yeast transformants carrying *oPAI* with molecular size at approximately 50 kDa (Figure 3b and 3c), which was consistent with the size of PAI. By comparison, the level of PAI expressed from the native *PAI* was extremely low and could not be detected by Western blot except for two transformants (Polh-1312-*PAI*-3 and Polh-1312-*PAI*-4, respectively) (Figure 3a). It was further observed that the level of PAI expression in multi-copy Polh-1292-*oPAI* transformants was significantly greater than that in the mono-copy *oPAI* transformants (Figure 3b and 3c). In addition, PAI expression level varied among individual transformants carrying the same expression cassette (Figure 3).

### PAI activity in *Y. lipolytica*

**Figure 4:**
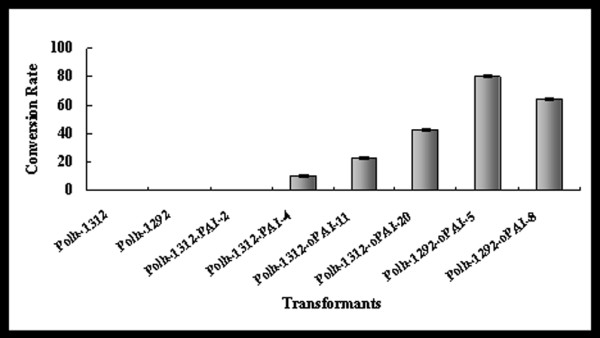
**Conversion rate of LA to CLA in selected*Yarrowia lipolytica*transformants.** Six represented transformants were selected for in vitro PAI activity assay. Polh-pINA1312 and Polh-pINA1292 were used as the control strains. PAI activity was represented by the conversion rate of LA to CLA, and calculated by the ratio of [CLA]/([LA] + [CLA]) × 100%.

In this study, the conversion rate of LA into CLA represented PAI activity. According to the PAI expression level, six represented transformants with best or average performance in each series were selected for in vitro PAI activity assay. In these selected transformants, PAI activities (Figure 4) was correlated with the expression level of PAI near linearly (Figure 3, relative density data was not shown). This phenomenon suggested that the ‘specific activities’ of PAI were comparable among the different transformants. The maximal conversion rate of 80% was obtained from transformant Polh-1292-*oPAI*-5. PAI activities were undetectable in transformants Polh-1312-*PAI*-2 and control strains (Polh-1312 and Polh-1292).

### Production of CLA in *Y. lipolytica*

**Figure 5:**
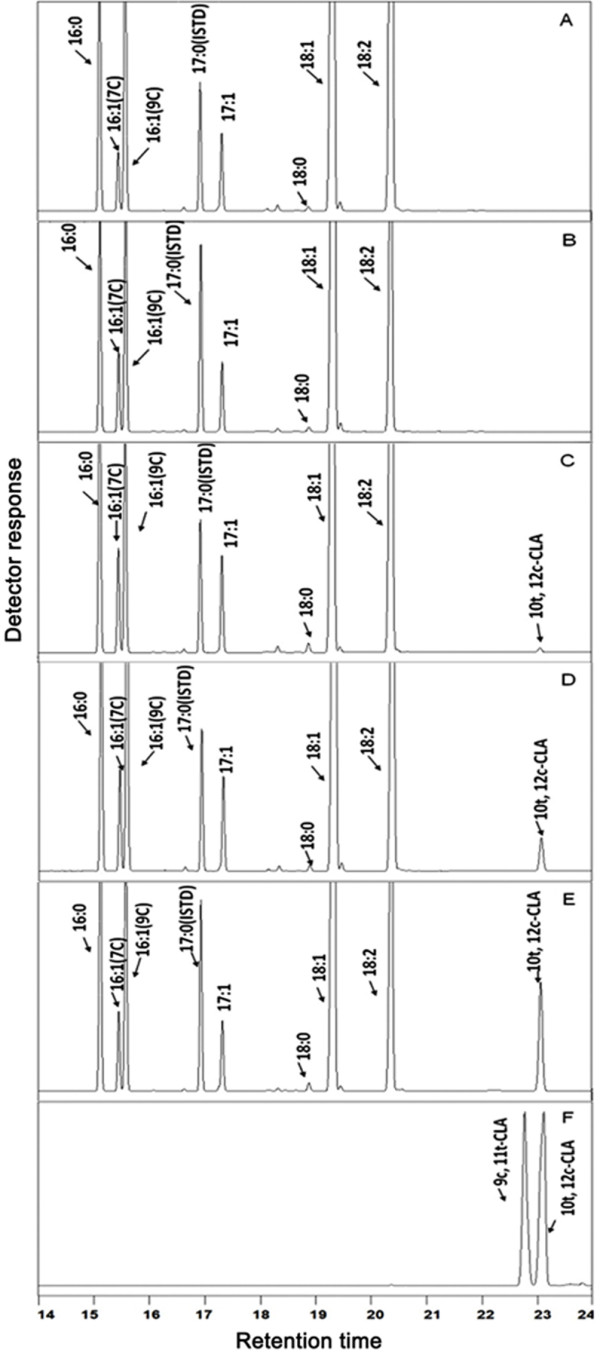
**Gas chromatogram analysis of FAMEs from transformed yeasts.** FAMEs from yeasts transformed with empty mono-copy vector pINA1312 **(a)**, empty multi-copy vector pINA1292 **(b)**, pINA1312-*PAI***(c)**, pINA1312-*oPAI***(d)**, pINA1292-*oPAI***(e)**, and commercial CLA mixture **(f)** were analysis by GC. Heptadecanoic acid (17:0) in the chromatograms was used as the internal standard (ISTD).

**Figure 6:**
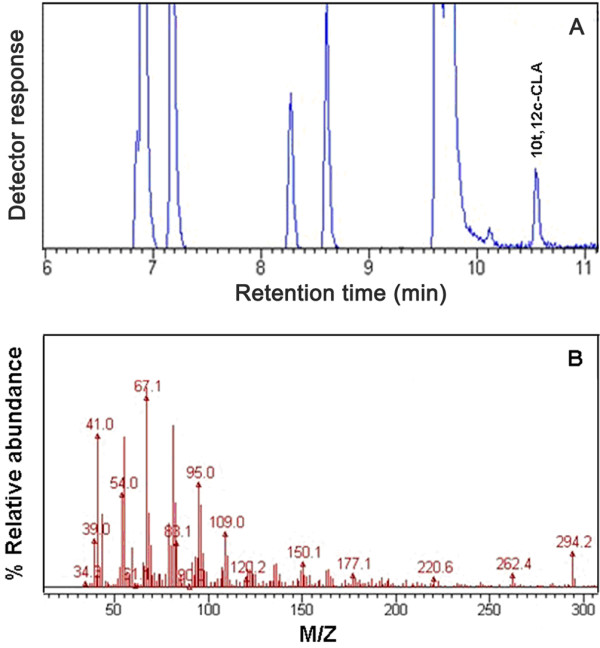
**GC/MS analysis of fatty acid isolated from yeasts transformed with pINA1312-*oPAI*.(a)** Shows gas chromatogram of FAMEs from Polh-pINA1312-*oPAI*. **(b)** Shows mass spectrogram of *trans*-10, *cis*-12 CLA at 10.5 min in gas chromatogram **(a)**.

To detect the possible production of CLA in the PAI transformants, lipid was extracted from these cells and FAME products of the lipid were prepared. FAMEs were qualitatively and quantitatively analyzed by GC and GC/MS. The results shown in Figure 5 and Table 1 revealed that there was no significant difference in the overall fatty acid composition between transformants and control strains except for a new peak, which was present in some transformants but not observed in control strains. The newly produced FAME was identified as *trans*-10, *cis*-12 CLA, comparing with authentic commercial standards. The identity of this CLA isomer was further verified by GC/MS (Figure 6).

**Table 1:** **Fatty acid composition of five transformed strains of*Y. lipolytica***

	**Polh-1312 (control)**	**Polh-1312-*PAI*-4**	**Polh-1312-*oPAI*-20**	**Polh-1292-*oPAI*-5**	**Polh-1292 (control)**
16:0	22.1 ± 0.5	9.1 ± 0.2	7.4 ± 0.1	10.9 ± 0.3	7.5 ± 0.3
16:1(7c/9c)	9.2 ± 0.2	10.2 ± 0.3	12.0 ± 0.3	12.7 ± 0.3	12.6 ± 0.5
17:1	1.9 ± 0.0	2.7 ± 0.1	2.3 ± 0.1	2.7 ± 0.1	2.5 ± 0.1
18:0	0.12 ± 0.0	0.3 ± 0.0	0.2 ± 0.0	0.4 ± 0.0	0.2 ± 0.0
18:1	42.4 ± 1.4	48.2 ± 1.5	51.8 ± 1.3	49.0 ± 1.1	59.1 ± 1.6
18:2	24.4 ± 0.6	29.2 ± 1.0	25.1 ± 0.7	18.5 ± 0.4	18.1 ± 0.8
10 t, 12c- CLA	ND	0.2 ± 0.2	1.2 ± 0.03	5.9 ± 0.1	ND

Values are represented at weight % of the total fatty acids of yeast transformants. The means ± SD were obtained from three independent experiments. ND, not detected.

In correspondence with the Western blot results, *trans*-10, *cis*-12 CLA was detected in all yeasts transformed with the optimized gene *oPAI,* but only in two transformants with the native gene *PAI* (Polh-1312-*PAI*-3 and Polh-1312-*PAI*-4). Besides, the CLA yields in individual strains with same expression cassette varied greatly as the PAI expression levels. The maximal amount of CLA in the yeasts transformed with native *PAI* was only 0.2% of total fatty acids (w/w). By contrast, the concentrations of CLA in all mono-copy *oPAI* transformants were above 0.2% with the maximal of 1.2%. Moreover, the average CLA contents in the multi-copy *oPAI* transformants were further increased to 3.4% and the maximal CLA concentration reached 5.9% (Table 1). Codon optimization of *PAI* resulted in a 6-fold increase in the maximal production of CLA. With multi-copy integration being taken into account, the improvement reached an approximately 30-fold increase over mono-copy *PAI* transformants. Taken together, our Western blot analysis, in vitro enzymatic activity assay and in vivo CLA yields analysis results demonstrated that the increased CLA yield was due to the enhanced expression level of PAI in these yeast transformants.

## Discussion

To date, the LA isomerase originating from *P. acnes* is the only LA isomerase that has been thoroughly investigated through biochemical characterization and crystal structure analysis, and appears to be of potential biotechnological applications. However, as the only substrate of PAI is free fatty acid, a minor component in most microbes and plants, it is difficult to produce sufficient *trans*-10, *cis*-12 CLA through genetic engineering of PAI. Several studies have reported to produce CLA by biological processes, through introduction of *PAI* into tobacco seed and rice, but the amount of CLA produced in these transgenic plants was only 0.3% and 1.6% of the total fatty acid amounts, respectively [12,13]. An appropriate biosynthesis system for CLA production has not been found yet.

To address this problem, *Y. lipolytica* was chosen as the host strain for PAI expression and CLA production in this study. *Y. lipolytica* is currently the only oleaginous yeast for which specific genetic tools have been developed and has ability to accumulate significant amounts of free LA [15]. Our results demonstrated that transformed *Y. lipolytica* could produce considerable amounts of CLA, and the production of CLA was increased with a multiple-copy expression of a codon-optimized gene.

In a previous study, PAI expression was improved in *S. cerevisiae* after optimizing the 20 N-terminal amino acid residues of PAI [12]. In our study, the full-length of the PAI gene was optimized, and as expected, codon-optimization fundamentally enhanced PAI expression (Figure 3) as the translation efficiency was promoted. These results demonstrated that the optimization of codon usage is important to enhance the expression of prokaryotic gene in a eukaryotic host organism. Multi-copy vector was also successfully used for the increased production of many proteins in *Y. lipolytica*, a 10 to 20-fold increase can be obtained over mono-copy integrants [18]. In comparison with the mono-copy plasmid, the amounts of expressed PAI in almost all multi-copy transformants were greatly increased due to the gene dosage effect (Figures 2 and 3). However, variation of PAI expression levels was also observed in different strains carrying similar copy numbers of the gene (Figures 2 and 3). A possible explanation for this phenomenon is that some integration sites of the expression cassette in the genome may affect the PAI production. Expression cassettes bound by the *zeta* regions have been found to randomly integrate in the chromosome of *Y. lipolytica* Polh lacking *zeta* regions [19]. This also indicates that it is necessary to screen as many transformants as possible to isolate the ideal transformants.

The combined effects of codon-optimization and gene amplification by using a multi-copy vector allowed us to increase the CLA production by almost 30 times, which is in agreement with the protein expression results. In the present work, we analyzed lipid compositions of all the transformants carrying the multi-copy cassettes. And the maximal CLA amount reached 5.9% of the total fatty acid yield in *Y. lipolytica*, which is much higher than that found in transgenic tobacco seed and rice [12,13]. The positive correlation between the protein expression level and CLA yield also suggested that the expression level of PAI was one of the key limiting factors for CLA production. Therefore, optimization of the cultivation conditions and genetic modifications will be carried out in the future to further increase the yield of CLA in *Y. lipolytica*.

## Conclusions

In this work, we have successfully constructed a *de novo* CLA biosynthesis system by transforming the oleaginous yeast strain, *Y. lipolytica*, with the recombinant linoleate isomerase gene from *P. acnes*. The expression level of PAI was dramatically increased by optimization of the codon-usage of *PAI* in the yeast and increasing the copy number of the gene using multi-copy vector*.* Coincide with the increased recombinant protein level, the *trans*-10, *cis*-12 CLA yield increased as well, with a 30-fold increase compared to the mono-copy integrants carrying native *PAI*. The best performed transformed yeast strain could produce up to 5.9% of CLA of the total fatty acid yield. The CLA yield in the transformed *Y. lipolytica* was much higher than those found in the transgenic non-oleaginous plants. Therefore *Y. lipolytica* should be considered as a CLA biological synthesis system of choice for future industrial applications.

## Methods

### Chemicals, strains and culture media

Restriction enzymes were purchased from Takara Bio, Inc (Takara, Dalian, China). The KOD plus DNA polymerase was purchased from Toyobo Co., Ltd. (Katada, Ohtsu-shi, Shiga-ken, Japan) and fatty acid methyl ester (FAME) standards were obtained from Sigma (Sigma, Shanghai, China). Oligonuleotide primers and other chemicals were supplied by Shanghai Sangon Biological Engineer Technology & Service Co., Ltd. (Sangon, Shanghai, China).

*E. coli* DH5α was used for routine subcloning and plasmid propagation. *E. coli* BL21 (DE3) was used for the over-expression of recombinant protein for antibody generation. The auxotrophic strain *Y. lipolytica* Polh [18] was used as the PAI expression host, while *Y. lipolytica* Polg was used as a control strain for real-time polymerase chain reaction (RT-PCR). *Y. lipolytica* strains were kindly provided along with the plasmids pINA1312 and pINA1292 [17] by Prof. Catherine Madzak (Institut National de la Recherche Agronomique/AgroParisTech, France).

All *E. coli* strains were grown in Luria-Bertani broth (LB) containing ampicillin (100 mg/ml) or kanamycin (50 mg/ml) for plasmid selection. YPD and YNBD media were prepared for *Y. lipolytica* as described previously [20].

### Plasmid construction, yeast transformation and gene expression

Standard protocols were followed for DNA manipulation [21]. In this study, the native and codon-optimized nucleotide sequences of PAI were cloned and expressed in *Y. lipolytica*. The native coding sequence (*PAI*) (GeneBank AX062088), flanked with *Pml*I and *Kpn*I, was amplified by PCR using the primers P1 and P2 (sequences see below) and template plasmid pGEX6-1-*PAI*, which was kindly provided by Prof. Ivo Feussner (Georg-August-University, Göttingen, Germany). Standard PCR consisted of 30 cycles of 15 seconds at 94 C, 30 seconds at 57 C, and 15 seconds at 68 C.

P1: 5'-CACGTGATGTCCATCTCGAAGG-3';

P2: 5'-GGTACCTTACACGAAGAACCGC-3'.

Underlined nucleotides depict the restriction sites. The codon-optimized sequence (*oPAI*) was designed according to the codon preference of *Y. lipolytica* using the Genscript OptimumGene^TM^ system. GCCACA was added before the first methionine to create a Kozak sequence. The newly designed DNA sequence flanked with *Pml*I and *Kpn*I was synthesized (Genscript, Nanjing, China). The two versions of DNA fragments digested with the appropriate restriction enzymes were ligated to form the mono-copy plasmids pINA1312-*PAI* and pINA1312-*oPAI*, respectively. The vector pINA1292, which carries a defective *URA3* allele, *ura3d4*, for multi-copy integration into the genome [17], was preferably used for PAI over-expression in *Y. lipolytica*. The construction of multi-copy plasmid pINA1292-*oPAI* was performed following the same procedures used for the case of pINA1312-*oPAI* described above. All newly constructed plasmids were screened by restriction enzyme digestion and PCR, and then confirmed by DNA sequencing.

Transformation was performed by the lithium acetate method, as described previously [20]. Transformants were selected by plating on YNBD. Real-time PCR (RT-PCR) was used to estimate the copy number of the integrated expression cassettes in Polh-1292-*oPAI* transformants according to the protocol described in [22]. *Y. lipolytica* transformants were grown in YPD medium at 28 C overnight. These pre-cultures were inoculated into 50 ml of YPD medium to an OD_600_ of 0.1 and further cultured for another 72 h at 28 C and 200 rpm. Cells were collected by centrifugation (6000 g; 5 min) and washed once with deionized water, and the pellet was either directly used for Western blot analysis and in vitro enzyme activity assay or lyophilized for biomass determination and lipid analysis.

### Generation of PAI-specific polyclonal antibodies and Western blotting analysis

His-tagged fusion PAI was produced in *E. coli* BL21(DE3) and purified by nickel ion affinity chromatography as described by Hornung et al. [12]. The purified His-tag-PAI was used as antigen for the generation of specific polyclonal antibodies in rabbits (AbMax Biotechnology, Beijing, China). To confirm the recombinant PAI expression in *Y. lipolytica*, Western blot analysis was carried out according to previously described methods [12].

### Lipid extraction and fatty acid analysis

Lipids from the equivalent weight of freeze-dried cells (50 mg) were directly transmethylated using the method described by Sakuradani et al. [23]. Fatty acid methyl esters (FAMEs) were analyzed by GC and GC/MS. GC analysis was performed with a GC-2010 (Shimadzu Co., Japan) equipped with a FID detector and a capillary DB-WAX column(30 m × 0.32 mm, φ0.25 μm; Agilent, USA). Helium was used as carrier gas (2 ml/min). The samples were measured with a split of 15:1 with the injector temperature set to 240 C. The temperature gradient was 120 C for 3 min, 120 C–190 C at 5 C/min, 190 C– 210 C at 1 C/min and 210 C for 3 min. FAMEs were identified by comparing with commercial FAME standards (37 Component FAME Mix, Supelco, USA; conjugated linoleic acid methyl ester, Sigma, USA) and quantified by the internal standard method by adding 146 μg of commercial C17:0 (Sigma, USA). GC/MS analysis was carried out using a Varian 1200 L GC/MS-MS equipped with a capillary DB-FFAP column (30 m × 0.25 mm, φ0.25 μm; Agilent, USA). Helium was used as carrier gas (0.8 ml/min). The temperature gradient was 180 C for 1 min, 180– 250 C at 5 C/min, 250 C for 21 min. The electron energy was set to 70 eV, and the ion source temperature and transfer line temperature were held at 200 C and 260 C, respectively.

### In vitro enzymatic activity assay

Yeast cell pellets were re-suspended in Tris buffer (100 mM Tris/HCl pH 7.5; 10 mM NaCl, 10% glycerol). The soluble protein from the yeast cells were obtained as described in [24]. A total of 8 mg soluble protein in 1 ml Tris buffer were incubated for 1 hour at 37 C with 800 μg LA. Fatty acid from the reaction system was extracted, methylated as described previously [9] and analyzed via GC described above. PAI activity was represented by the conversion rate of LA to CLA, and calculated by the ratio of [CLA]/([LA] + [CLA]) × 100%.

## Abbreviations

CLA = Conjugated linoleic acid; LA = Linoleic acid; PAI = Linoleic acid isomerase originating from Propionibacterium acnes; FAME = Fatty acid methyl ester.

## Competing interests

The authors declare that they have no competing interests.

## Authors’ contributions

BXZ carried out the experiments and drafted the manuscript. CCR participated in lipids extraction and data analysis. HQC and WC participated in the experimental design and reviewed the manuscript. YDS and HZ conceived the study and reviewed the final manuscript. All authors read and approved the final manuscript.
